# Supplementary material for: Modulation of prion protein expression through cryptic splice site manipulation
Source: J Biol Chem. 2024 Jul 11;300(8):107560. doi: 10.1016/j.jbc.2024.107560 (PMC11342779; doi:10.1016/j.jbc.2024.107560)
Supplement: Supporting Data 2 [file mmc2.pdf]

SUPPORTING INFORMATION

Modulation of prion protein expression through cryptic splice site manipulation

Juliana E. Gentile[1,2], Taylor L. Corridon[1,2], Meredith A. Mortberg[1,2], Elston Neil D'Souza[3], Nicola Whiffin[3,4], Eric Vallabh Minikel[1,2], Sonia M. Vallabh[1,2]

Supplementary tables 1-6 provided as a separate Excel file.

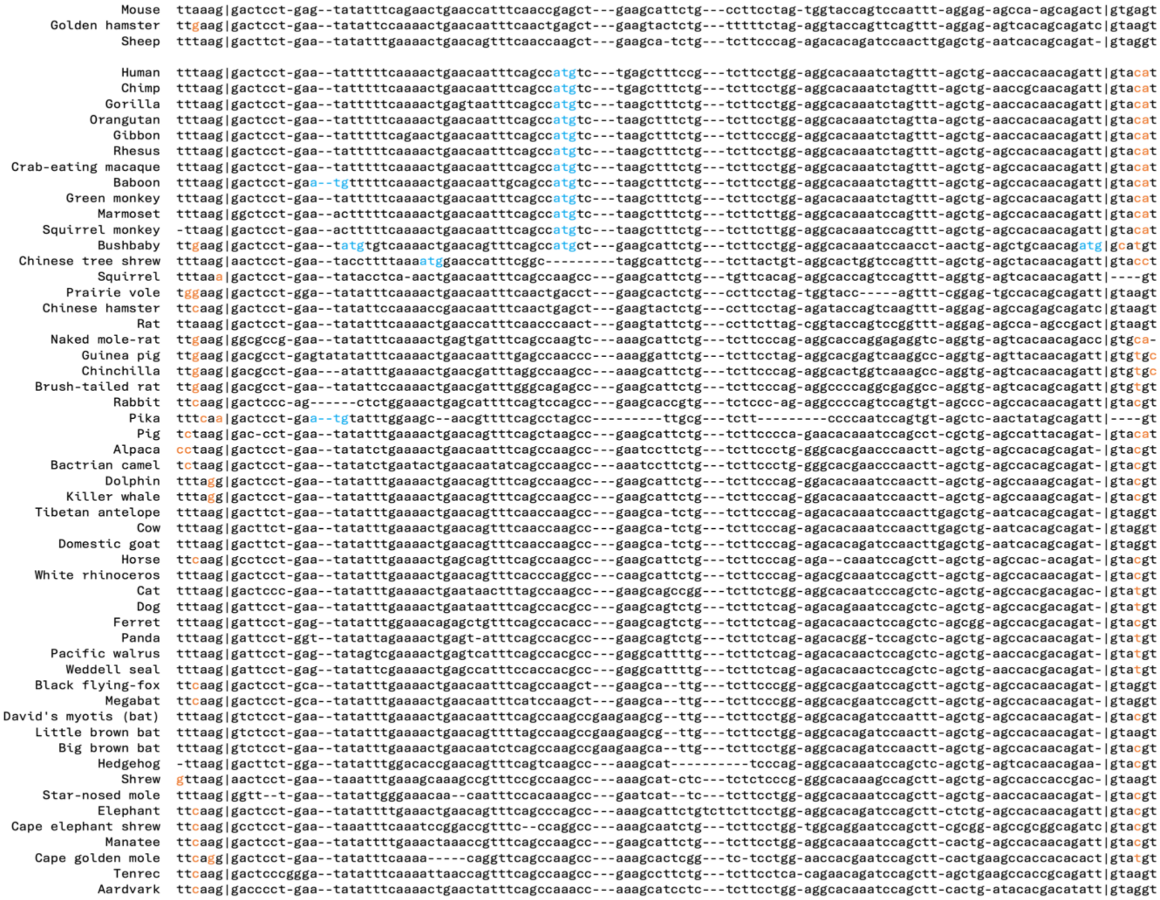

Figure S1. Multiple alignment of PRNP exon 2 orthologous sequence for all available eutherian mammals. *As in Figure 1D, but including eutherian mammals without ATGs in exon 2. Lesser Egyptian jerboa is excluded because orthologous sequence was identified for only part of exon 2.*

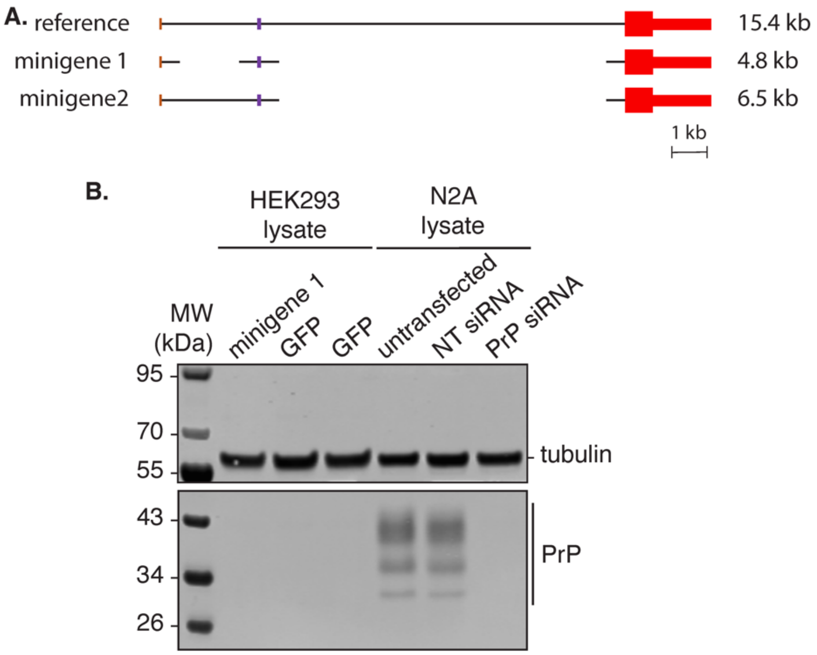

Figure S2. Alternative minigene construct tested in cells. A) *Comparison of human reference sequence with an alternative "minigene 1" containing only 500 bp at either end of intron 1, and the "minigene 2" used throughout the main text of this manuscript.* ***B)*** *Immunoblot failing to detect any expression of minigene 1 in transfected HEK293 cells. Primary antibody: 6D11, see Methods.*

	**exon 2 splice sites**<br>**acceptor** **donor**
**template (wild-type)**	TTTAAG|GACTCC...TCAGCCATGTCTGAG...CAGATT|GTACAT
**canonical splice site (ss)**	TTTCAG|GACTCC...TCAGCCATGTCTGAG...CAGCAG|GTAAGT
**canonical ss- ATG mutant**	TTTCAG|GACTCC...TCAGCCCCCTCTGAG...CAGCAG|GTAAGT
**mouse splice site (ss)**	TTAAAG|GACTCC...TCAGCCATGTCTGAG...CAGACT|GTGAGT
**mouse ss- ATG mutant**	TTAAAG|GACTCC...TCAGCCCCCTCTGAG...CAGACT|GTGAGT

Figure S3. Variants with the exon 2 uORF abolished. *As in Figure 3A, but with additional mutants replacing the ATG with CCC.*
